# Processing Speed Accounts for the Discriminative Power of a Smartphone Go/No-Go Task in Multiple Sclerosis

**DOI:** 10.64898/2026.09.09.26362640

**Published:** 2026-09-11

**Authors:** Peter Kosa, Amir Moghadam Ahmadi, Marie Kanu, Yolanda Mejia, Emmanuel Mekasha, Caledonia Steltzner, Bibiana Bielekova

## Abstract

**Background:**

Identifying and longitudinally measuring cognitive decline remains challenging because traditional assessments rely largely on episodic, in-person administration of analog scales by trained examiners. Smartphone technology provides a scalable alternative capable of extracting distinct digital biomarkers from continuous sensor and event streams. Following the integration of several cognitive modules into the self-administered Neurological Functions Test Suite (NeuFun-TS), this study evaluates the clinical utility and incremental validity of a final candidate module: an equiprobable (50/50) continuous vigilance Go/No-Go paradigm.

**Methods:**

We analyzed 3,270 quality-controlled trials from 303 participants, focusing primary analyses on 488 first-trial observations across 34 healthy donors (HD) and 213 multiple sclerosis (MS) participants. From the raw event stream, we screened 16 candidate metrics and retained 10 based on their intrinsic psychometric properties. Using these 10 metrics, we evaluated diagnostic group separation, test-retest reliability, correlations with clinical and magnetic resonance imaging (MRI) benchmarks, and incremental validity over existing digital modules: the randomized Symbol Digit Modalities Test (rSDMT) and the Motor Sequencing Test (MST), using paired observations.

**Results:**

Although nine of the 10 outcomes significantly separated HD from MS and seven separated relapsing-remitting from progressive MS subtypes (all adjusted p < 0.05), discriminative power was driven almost entirely by response latency rather than commission errors or inhibitory metrics. Multi-predictor composite scores offered no diagnostic advantage over reaction time alone. Furthermore, the Go/No-Go task demonstrated poor-to-moderate longitudinal test-retest reliability and failed to provide incremental clinical value over existing modules.

**Conclusion:**

Although the smartphone-based Go/No-Go task reflects processing speed impairment in MS, its discriminative capability is redundant with simpler reaction time metrics already implemented in the platform. Consequently, the Go/No-Go module was excluded from NeuFun-TS to minimize patient testing burden without compromising diagnostic sensitivity.

## Full Text Availability

The license terms selected by the author(s) for this preprint version do not permit archiving in PMC. The full text is available from the preprint server.

